# An Approach to the Impact Simulation on Foamed Injection Molded Polypropylene Parts: An Example of Application in the Automotive Industry

**DOI:** 10.3390/polym15040936

**Published:** 2023-02-14

**Authors:** Massimo Nutini, Markus Franzen, Mario Vitali

**Affiliations:** 1 Basell Poliolefine Italia Srl, LyondellBasell Industries, p.le Donegani 12, 44121 Ferrara, Italy; 2 Basell Sales & Marketing Company B.V., LyondellBasell Industries, Huettenstr. 130–138, 50170 Kerpen, Germany

**Keywords:** polypropylene, foam, impact, finite element analysis, automotive, lightweighting, occupant protection

## Abstract

An approach to the simulation of foamed injection molded Polypropylene parts subjected to impact loading is presented in this paper. The proposed method, which considers strain-rate-dependent material properties and the possible occurrence of fracture, is, in particular, suitable for parts manufactured with core-back technology. The method was developed to be used within the functionality of a commercial Finite Element solver using a shell-type element mesh. The material model is based on a three-layer structure, with two compact skin layers and a foamed core layer made of expanded material. The properties of the foamed material are assumed as those of the compact grade scaled by a suitable factor, which is identified via inverse engineering on a set of bending tests executed on specimens having different foam densities. The fracture of the material is then predicted using a damage model which considers the effects of triaxiality. The approach is then validated on industrial parts from the automotive sector, subjected to impact in a component test. Despite the simplicity of the presented approach, which makes this method suitable for industrial applications and especially for early-stage design, the validation shows a sufficiently accurate simulation of part behavior under the impact, with a reasonable prediction of damage and fracture.

## 1. Introduction

As part of the trend toward a circular economy [1], the trend towards lightweight materials has become a fundamental strategy in the transportation industry in recent years since it is recognized that transportation accounts for a significant portion of the total energy consumption [2]. This has become even more important in regard to the rapid transformations occurring in the automotive industry, including electrification and autonomous driving, where the reduction of vehicle weight mirrors significant improvements in fuel economy [1,3].

In the search for lightweight materials and structures, an interesting opportunity is given by the use of sandwich structures [1], in particular with polymers. Polypropylene (PP) sandwich structures with foam core have attracted much attention due to their advantage in terms of high strength-to-weight and high stiffness-to-weight ratio, not only in the transportation industry but also for other applications [4]. Limiting the attention to transportation, for example, in [5], an application of PP foam on the door panel of the Toyota CH-R SUV is shown, with the goal of reducing the weight and the injection cycle time in its production.

Simulation-driven techniques [1,2] play a crucial role in the development of light weighting strategies. With their help, the design of structures can be optimized with regard to a minimal use of material and better material distribution. It is therefore of great importance that reliable and efficient numerical models for the description of material behavior are developed, and computational material science and ICME (Integrated Computational Materials Engineering) are receiving increased attention, especially when coupled with Finite Element methods, which are the recognized tools for the efficient design of parts and structures. In this context, the methodology we developed, including the material described as a sandwich structure, aims to provide a computational method for parts made of injection-molded and expanded PP. In this type of process, foaming can be achieved through physical or chemical means. Chemical foaming is often preferred since it does neither require expensive modifications to the equipment nor license fees. 

Parts produced with this process are characterized by a structure with two compact skins and a foamed core in between [6,7] so that a sort of sandwich structure is formed. Differently from foam structures obtained with other processes, in this case, a clear distinction between the different layers is not observed [6], but layers can be more or less distinguishable depending on the process parameters, and in particular depending on the cooling conditions, on the temperature differences between the mold and the melt and on the amount of Chemical Blowing Agent (CBA).

A possible embodiment of this process is the so-called “Breathing Mold” or “Core Back” technology, by which the pressure drops in the mold cavity, needed for the material to foam, are ensured by the opening of the mold, which is obtained through the movement of the mold wall.

Several authors have investigated the mechanical properties of parts produced by injection molding processes and their relationship with material structure and foam morphology; some authors have also addressed computational models for describing such behavior.

Akkoyun et al. [8] investigated the effect of process parameters on the mechanical properties of foamed PP parts produced with the core back injection molding process in combination with CBA. They showed that increasing the injection speed will lead to an increase in the cell diameter. They also showed that cell diameter initially decreases with increasing core back distance but then decreases after the core back distance overcomes a critical value due to coalescence and collapse of cells that continue to grow. This is recognized as influencing mechanical properties. Kastner et al. [9] examined the influence of seven process parameters within a physical foaming process on the mechanical properties of the parts produced, thereby identifying the three factors that mostly affected the mechanical performances.

Çakir et al. [5] also observed that the mechanical properties and weight of the part are affected by cell morphology, which results from process parameters and, in particular, from CBA content. Yetgin et al. [10] showed the influence of process conditions on the morphological and mechanical properties of microcellular polymer materials in a study on more than 90 PP and PP + talc samples obtained varying the process parameters.

Sadik et al. [7] studied morphology and mechanical properties of samples from parts obtained with a core-back injection molding process. They measured the geometrical dimensions of the sandwich structure of the specimens and verified the accuracy of predictive models. A similar approach can be found in [11], where it is shown that mechanical properties in a sandwich structure with foamed core are also affected by the density profile. They investigated the effect on the flexural modulus of six different density profiles at constant average density.

Some authors developed computational methods to model the stress-strain behavior of foamed Polymers. Daniel et al. [12] focused on the strain rate effects on the mechanical performances of foam materials, developing a master stress-strain curve referred to one reference strain rate and introducing the strain-rate effect through a single parameter. However, they do not consider the possible dependence of material properties on the foam structure, which was instead assumed and not considered as a variable. Goga et al. [13] proposed a new constitutive model for cellular solids and a strategy for the determination of its parameters, which describes the foam stress-strain curve from compression tests as composed of an initial linear elastic region followed by a plateau region and then by a strain locking region. The model was tested on polyurethane and aluminum foams of various densities but did not take into account the strain rate effect. Numerical tools were also used to better understand how morphology influences the mechanical properties of the foam. Wei Gong [14] et al. showed, through a finite element analysis, that the reason for lower tensile and impact strength characterizing foamed structures with larger cells or with wider cell size distribution is the higher stress concentration. They demonstrated that voids in the structure could be regarded as notches in the samples under tensile loading. 

From the examples reported above, it is clear that foamed materials pose a relevant challenge to the designers that intend to use such materials in their projects, as the workflow commonly used for Finite Element simulations cannot be applied: material properties are strongly process-dependent and, due to this fact, they cannot be simply obtained from the material provider. Additionally, any simulation activity must use dedicated computational models to consider the complexity of the material structure. Hence, simulation approaches to be used in the design of foamed parts and in the prediction of the behavior under impact loading are scarcely available in the literature, with only a few exceptions [15]. One purpose of the present paper is to fill this gap by addressing a methodology that could be used in the design of foamed parts. The main challenge for modeling this class of materials is clear from the considerations above: the properties of the material depend on its morphology which depends on the process conditions. Hence, material properties cannot be measured “a priori” with respect to the process, as it is usually carried out for the finite-element analysis of parts. Data cannot be provided by a polymeric material producer since the CBA, and its transformation in the process can affect the final mechanical behavior. Furthermore, the material description must anyway take into account the properties of the base polymer, or matrix, as how they depend on temperature and strain rate.

Finally, to predict failure or rupture, the effect of bubble density, bubble size, and base polymer damage behavior under loading must also be considered. It is a very complex physical behavior; we propose a phenomenological approach for a method to be practically used in the design of parts and, in particular, in an early design phase. This approach is suitable with the resources typically available in the industry in regard to commercial finite element codes and basic testing capabilities as well. For the activity reported here, the Finite Element solver LS-DYNA by LSTC was used [16].

The approach that is presented here is based on a sequence of steps:

1. The material is assumed to take, as the effect of molding and expansion, a three-layer sandwich structure, where the outer skins are compact, and the core is expanded. This structure is finalized to be transposed into a shell element-based finite element model.
2. Tensile tests at different speeds are executed on unexpanded specimens to measure the strain-rate-dependent mechanical properties of the compact material.
3. Bending tests at different speeds are then conducted on specimens with different expansion ratios to characterize the behavior of the foamed material.
4. The properties of the foamed material are defined, at different degrees of expansion, through a strain-rate dependent coefficient which reduces the properties of the compact grade as a function of the expansion.
5. Such scaling coefficient is identified through the finite element simulation of the bending tests on specimens mentioned at point 3, modeled as per point 1.
6. Areas of similar degree of expansion of the core, as resulting from local different final thickness after core-back mold opening, are associated with material properties scaled of the same scaling term as determined at point 3 above and hence associated to a properly scaled material law.
7. A damage and rupture criterion are set based on the foam structure.

After the sequence of operations described here, the finite element model is available for the simulation of the impact loading on a generical component, according to a procedure that is commonly followed in the finite element design practice. 

The following section will cover in detail the steps regarding the preparation of the computational model, with insight into the preparation of the material data and with additional considerations about damage and fracture modeling. Furthermore, details will be first given about the industrial part of automotive, which was chosen to validate the approach.

## 2. Materials and Methods

### 2.1. Industrial Part for Method Validation

The target for the presented activity is to provide a tool that could help the designers of components subjected to impact, with particular reference to the automotive sector, where foamed parts can be useful to reduce car weight. Accordingly, the component chosen to assess the validity of the approach proposed here was a car interior trim, as such components are likely to be involved in impact scenarios [17,18]. An industrial, automotive part, for which the mold was available, was then used within this study, with approximate dimensions of about 800 mm × 300 mm, having an unexpanded thickness of 2.0 mm. The choice of the material, which is in detail described in the following paragraph, was then for a grade developed for this kind of application. The part was injection molded with core back technology using a commercial endothermic CBA, resulting in exemplars with a final thickness of 2.4 mm, 3.0 mm, and 3.2 mm for component testing, and in additional exemplars with 2.8 mm thickness, used for material parameters determination.

Regarding component testing for method validation, the first activity was aimed at the design and definition of the physical experimental test set-up, defining the supports, the clamping, and the impact conditions so that the impact causes significant material deformation, damage, and possibly rupture. This preliminary study, which was conducted with finite element simulations using material characteristics of the compact grade, led to the experimental configuration being effectively used. 

The part was fixed with four screws on a support, which mimics the shape of the part itself, onto which the part is laying. In order to allow the support and part-facing surfaces to match, the support was manufactured with 3D printing technology to reproduce the geometry of the part and ensure a suitable contact. Additionally, plaques were screwed to the part on the opposite side with respect to the support so that the part was sandwiched between the support and plaques. As for the support, the surface of such additional plaques mimics the part geometry in order to establish continuous contact between the surfaces. 

The part was then subjected to a dynamic drop test. In all the cases, the impactor was mounted on a sleigh. The mass of the impactor was 20.7 kg, and the mass of the sleigh was 3.5 kg. Two types of impactors were used for testing. In a first embodiment, an impactor with a 20 mm diameter hemispherical head was used, with an impact speed of 3 m/s. In a second embodiment, the head of the impactor was hemispherical with a diameter of 65 mm. In this case, tests were conducted at two different impact speeds, namely 2 m/s and 4 m/s. All the tests were carried out at room temperature. 

### 2.2. Materials and Experimental Test for Characterization

The material used for this study was a talc-filled, impact-modified PP compound developed by LyondellBasell Industries for foaming applications, offering a good appearance, required melt strength, high impact performance at low temperature, and combining low odor and low emissions performances. The same material was used to mold the industrial parts for component testing, which were also used to extract the specimens for the bending tests, and to mold additional plaques, which were mainly used to extract specimens for the tensile tests finalized to the determination of the mechanical properties of the unexpanded grade.

These latter tests provided engineering stress-strain curves at 23 °C for nominal strain rates ranging from 0.01 s^−1^ to about 10 s^−1^. The curves were extrapolated using the Eyring assumption up to a strain rate of about 100 s^−1^. The main properties measured on the compact grade at different strain rates are reported in Table 1. 

For determining the material properties of the foamed parts to be used in the numerical simulation according to the procedure that is described in the following paragraph, specimens shaped as stripes of 100 mm × 10 mm were cut from the automotive molded parts and then subjected to three-point bending tests. These tests were conducted according to standard ISO 178 with speeds of 0.1 mm/s (quasi-static), 1 m/s, and 4 m/s; for all the tests, the extremities of the specimen were left free, thus allowing them to slide on the supports. The bending tests at higher speeds were executed with a pendulum. The width of the specimen was further milled to about 8.15 mm to reduce irregularities in their shape due to the previous cut; the span was 50 mm for all tests. The nominal stress *σ_B_* under bending was computed from the well-known formula

(1) $$\sigma_{B}=\frac{3\cdot L\cdot F}{2\cdot w\cdot s^{2}}$$

where *L* is the span, *F* is the measured force, and *w* and *s* are the width and thickness measured on the sample. The results are reported in Table 2.

### 2.3. Computational Model

**Figure 1:**
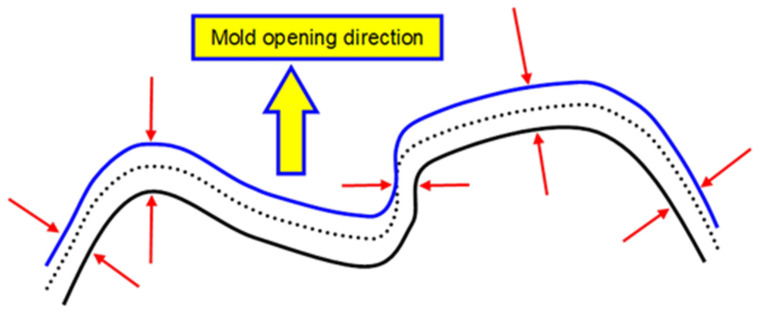
Mold opening in the core-back process causes an increase in the part’s local thickness, which locally depends on the mutual orientation between the part and the direction of the mold opening (thin red arrows indicate the local direction of the normal to the surface).

For reasons of computational efficiency, the model is designed for shell elements. The approach to the structural analysis of foamed PP developed by the authors and used for the present activity was already presented in previous publications [19], although limited at that time to static analysis. The main features will be here quickly reprised. All these features are available within the chosen solver. The model is suitable for foamed PP parts produced either with CBA or with physical foaming. To take into account the peculiarities of the core back technology, the structural mesh for Finite Element analysis is obtained from the mesh of the unexpanded part, taking into account the effect of the mold expansion on the local part thickness. The element thickness is hence modified as a function of the mutual orientation between the normal to the mesh element and the direction of the mold expansion, assuming said the expansion is proportional with respect to the cosine of the angle between the two directions. In other words, elements perpendicular to the core back direction experience the highest expansion, while elements parallel to the core back direction are not subjected to thickness variations. Figure 1 is a sketch that clarifies how thickness is changed depending on the local orientation of the part.

**Figure 2:**
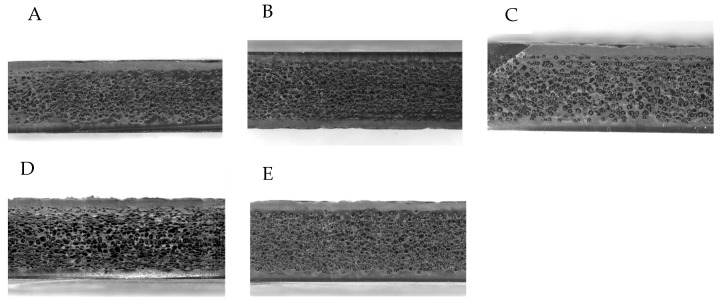
Structure core/skin in specimens cut from the core-back injection molded part. (**A**–**C**): specimens cut from the same position on the part, from parts 2.8 mm, 3.0 mm, and 3.2 mm, respectively. (**D**,**E**): Specimens cut from 3.2 mm thick parts from other different positions.

As previously remarked, when foaming technology based on an injection-molding process is used, a three-layer sandwich structure is formed [6,7], with “skin” layers of compact material in proximity of the mold surface and an expanded “core” layer in between, as shown in Figure 2. The images, all taken with a Zeiss Stereo Discovery Zoom V20 Optical Microscope, clearly show the structure core/skin in all the specimens cut from the industrial part considered, molded with different expansions.

**Figure 3:**
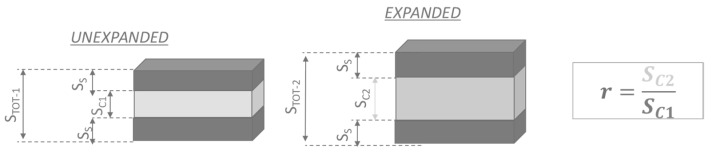
Three layers material model and definition of the “expansion ratio”.

Accordingly, in our model, the material in the skin layers is considered in the first approximation as one of the unexpanded grades. The skin thickness, which ultimately depends on the process parameters [6,7,8], is approximately uniform throughout the whole part. Common values for the skin layer thickness are about 0.30 mm to 0.60 mm [7]. For the parts molded here, a skin layer thickness of 0.45 mm was considered based on the image analysis of the specimens cut from the molded parts. The mechanical properties of the core are assumed to be derived from those of the unexpanded material through scaling coefficients, which expresses a sort of deterioration of the material due to the generation of bubbles on the inside. It is assumed that such deterioration may be a function of the local expansion and the strain rate. The parameter used for the definition of the degree of expansion is named “expansion ratio” and is defined as the ratio of the core thickness *Sc*_2_ in the expanded part to the core thickness *Sc*_1_ in the unexpanded part. This is explained with the aid of the sketch in Figure 3.

**Figure 4:**
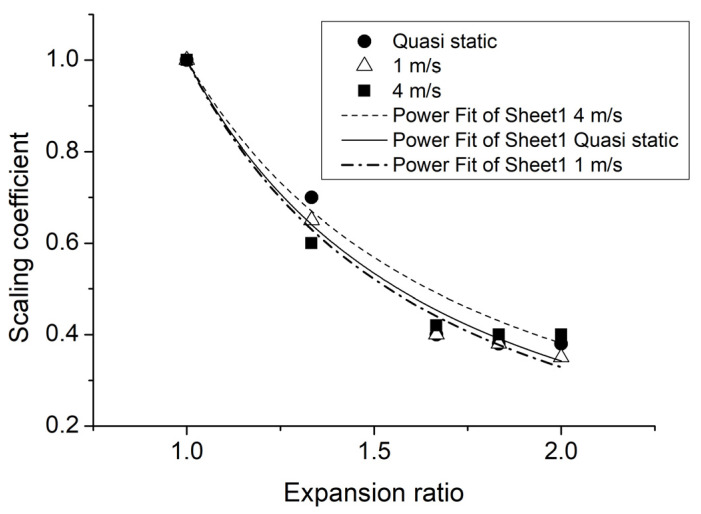
Coefficient for scaling the properties of the compact grade to obtain those of the expanded material as a function of the expansion ratio.

The scaling coefficients are computed through Finite Element in an approach as “inverse problem”, as the values that best reproduce bending tests carried out on specimens with different expansion ratios and tested at various speeds. As visible from Figure 4, the result of this calibration is a set of points on a surface which expresses how such scaling terms vary with expansion ratio and strain rate. An analytical function is then used to interpolate those points to facilitate the implementation in the Finite Element solver. It is, in fact, known to those familiar with Finite Element simulations that elements are usually grouped in sets, often referred to as “sections”, which collect elements with similar characteristics to which common properties (e.g., material, thickness) are assigned. Depending on the complexity of the problem, the user may choose how many of said “sections” are needed. An analytical form for material properties allows the computational engineer to choose the desired number of such “sections” without being limited by the number of experimental tests effectively carried out. Figure 4 shows only a minor effect of the strain rate. It must be emphasized once again that this is related to the specific foam structure obtained in this process, while the curves and parameter sensitivity may differ in the case of the use of different process parameters even with the same base material.

The material law “MAT_024” available in LS-DYNA was used [16]. This law, based on Von Mises Plasticity, is the most commonly used law in the automotive industry. MAT_024 is stable, reliable, and quick and offers, in general, a valuable tool for the designer. It accepts strain-rate-dependent material properties, which are easily introduced as tables. The material law parameters in the elastic domain are mainly Young’s modulus, the density, and Poisson’s ratio. For plasticity, a stress vs. plastic strain curve is the only input, as the material law does not differentiate between tension, compression, or other loading states. Accordingly, stress-strain curves from tensile tests were given as input, and each curve is associated with a specific strain rate, letting then the code interpolate between the assigned strain rate values to determine the properties of the actual strain rate. For the sections comprising unexpanded elements, the material properties were those measured on the compact grade. However, further tuning was carried out in that the simulation of the bending tests on specimens cut from parts produced with zero core back distance was used to correct the stress-strain curves measured on the compact grade to take into account the possible influence of the presence of the CBA in the material. For the “sections” with expanded core, Young’s modulus, the density, and the stress-strain curves of the material in the core were those of the compact grade multiplied times the scaling coefficient determined as per the above.

### 2.4. Damage and Fracture

**Figure 5:**
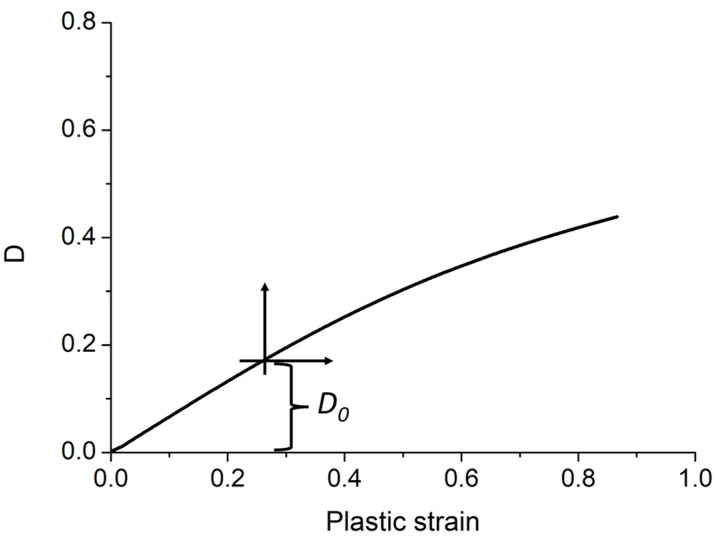
The pre-damage *D*_0_ derived from the tensile tests is subtracted from the ultimate damage measured at failure for the compact material to obtain the effective limit damage leading to rupture.

Damage occurring in the material is assumed to lead to material failure. For this study, the approach to damage follows the one of Chaboche–Lemaitre [20], by which damage is associated with the creation of voids inside the material so that the stress between nearby portions of the material is exchanged through a reduced area or “effective” area. Mathematically, the damage parameter is simply the fraction of the cross-sectional area occupied by the voids, ranging from 0 (undamaged) to 1 (fully damaged). In previous studies [21], Nutini et al. introduced a methodology for measuring the damage according to Chaboche–Lemaitre on a solid polymer subjected to a tensile test based on the measurement of the volume variation. Hence, the damage, intended as the fraction of the cross-sectional area occupied by the voids, was derived from local strain measurement. The mentioned methodology was also used by other research teams [22], which also demonstrated that such a method leads to results comparable to those achieved by methods based on the loss of stiffness [23], but in a more effective and easy way [24]. For the present study, the main concept onto which failure modeling is grounded is that the foam structure may be regarded as a “pre-damage” *D*_0_ on the material in that the bubbles in the material play the role of Chaboche–Lemaitre voids. Accordingly, the evolution of the damage was supposed to follow the damage curve measured on the compact material sample. The material damage leading to rupture was then measured on specimens of the unexpanded material subjected to tensile test up to rupture. Furthermore, the initial pre-damage, assessed from the foam structure, provides in this view only a shift in the damage curve, making the path to failure shorter. In Figure 5, an example is shown of how said shift in the origin of the damage curve measured on the compact grade is here used to consider the initial foam structure. This is clearly an approximation since the damage mode of the foamed material under bending—which is the typical working condition for foamed parts—may evolve in general with different modalities. The assessment of the initial pre-damage was based on results from tensile tests executed on samples with different expansions, taking advantage of the fact that methods based on loss of stiffness provide comparable results to those based on volume variation [22], which in this case allow using the formula:

(2) $$D_{0}=1-\frac{E}{E_{C}},$$

*E_c_* is the modulus measured on the compact grade, and *E* is the modulus of the foamed grade.

The damage curve has been introduced in the LS-DYNA solver through the model therein available, known as “GISSMO” [16,25]. The main concept on which “GISSMO” is based is the accumulation of damage, where the damage rate depends on the loading conditions: these are expressed by the triaxiality parameter, namely the ratio of the pressure to the von Mises equivalent stress. Once a damage threshold is reached, the material degrades its properties till it is no more effective in sustaining any loads. Accordingly, a “GISSMO” card must provide information on the damage evolution vs. triaxiality. The damage curve used for this purpose is described in the solver in analytical form, which is scaled by triaxiality-dependent coefficients. The analytical form expressing the evolution of damage with respect to the plastic strain is:

(3) $$\Delta D=\frac{n}{\varepsilon_{f}}D^{\left(1-\frac{1}{n}\right)}\cdot \Delta \varepsilon_{p}$$

where *D* is the damage, *n* is the fading parameter, *ε_f_* is the failure strain, and *ε_p_* is the plastic strain. Both parameters *n* and *ε_f_* were determined by fitting the damage curve measured as described before. For the specific material here considered, parameter identification on the damage measured curve led to *n* = 0.95 and *ε_f_* =1.8, while the threshold damage was assessed as about 0.45, which was measured from the test at 100 mm/s. 

The effect of the triaxiality on the failure strain *ε_f_* was input through a table, indicating for each triaxiality value a factor that the software uses for scaling the failure strain associated with tensile loading as determined before. In the absence of experimental data, generical values have been taken from the literature. Regarding shear loading, a reference was made to Dayan et al. [26]. For a 20% talc-filled, impact-modified PP, a reduction in elongation at break from 0.6 to 0.35 was associated with variations in triaxiality from 1/3 (tensile loading) to 0 (biaxial loading). The same proportions were kept for the present study. Regarding compressive loading (triaxiality values −0.33 for uniaxial and −0.66 for biaxial compression), the scaling coefficients were kept quite high in order to avoid failure under compression. Regarding biaxial tensile loading (triaxiality value 0.66), reference was made to the papers by Lin et al. [27], where tests were conducted on notched specimens exploring triaxiality values of 0.4 and 0.66, obtaining values of elongation at break slightly lower than those achieved under tensile loading. For the present study, it was decided to assume elongation at break under high values of triaxiality as half of the one measured under uniaxial tensile loading. Finally, the last parameter considered in the “GISSMO” card was a variable (named “FADEXP”) which controls the stress fading after rupture. A very small value, which determines element failure as soon as the critical damage at rupture is reached, was chosen. The resulting parameters for the “GISSMO” card are summarized in Table 3.

## 3. Results

**Figure 6:**
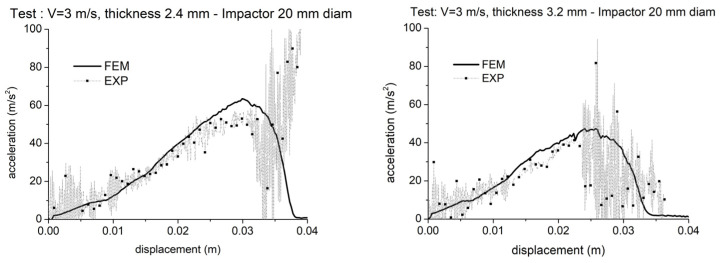
Force—displacement curves measured using the 20 mm-diameter hemispherical impactors: impact speed 3 m/s, nominal part thickness 2.4 mm (**left**) and 3.2 mm (**right**).

Impactor force-displacement curves measured in two drop tests with the 20 mm head impactor on industrial parts with different expansion ratios are displayed in Figure 6 in comparison with the curves obtained from simulations. The Finite Element model used for all the simulations presented here was set up to reproduce as close as possible the physical, real test boundary conditions.

Both tests show a good agreement between experiment and simulation; the force values are correctly reproduced, and the occurrence of fracture is captured by simulations in correspondence with the correct impactor displacement.

**Figure 7:**
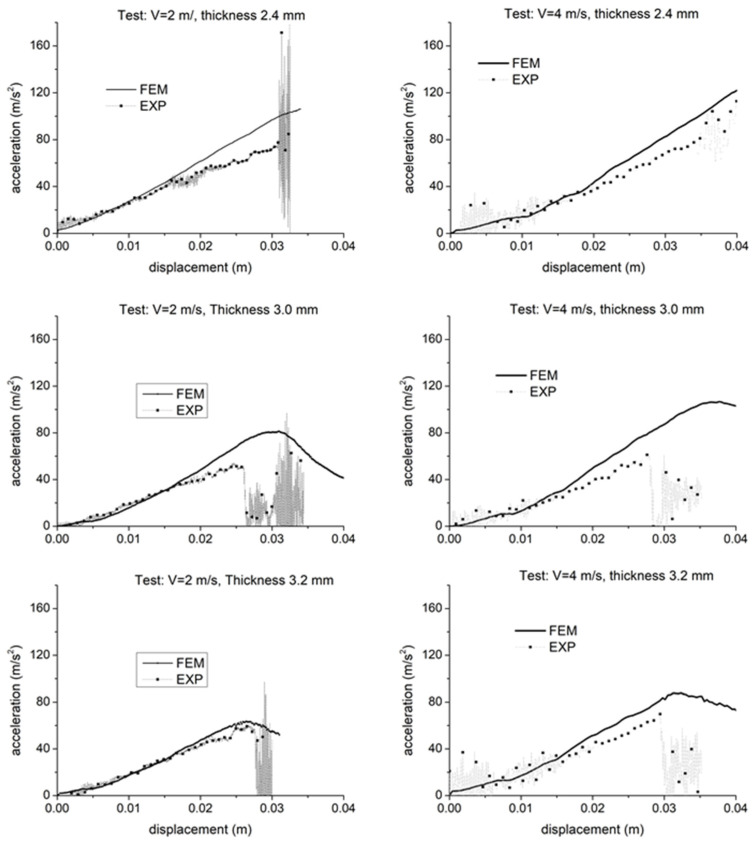
Force—displacement curves measured using the 65 mm-diameter hemispherical impactors: impact speed 2 m/s (**left**) and 4 m/s (**right**), nominal part thickness 2.4 mm (**top**), 3.0 mm (**central**), 3.2 mm (**bottom**).

Referring to the tests carried out with the 65 mm head impactor, the experimental force-displacement curves measured under different experimental conditions with thickness from 2.4 mm to 3.0 mm and impact speed from 2 m/s to 4 m/s were reasonably reproduced by the simulations. Reference is made to Figure 7. The cases examined here show a generally good agreement between the simulation results with the experimental curves. In particular, the initial part of the curve is well captured in all the cases. Some discrepancies are, however, observed for higher strains, in particular for the cases with lower expansion ratios. For all the expansion ratios, the simulations show stiffer behavior than in the physical tests. Forces always exceed the experimental values. The reason for this deviation will be discussed in the following paragraph. In the graphs, fracture in simulation occurs in correspondence with a sharp change in the slope of the acceleration curve, precisely at the peak related to the maximum acceleration. Except for the graph on the top left in Figure 7, which is related to a test where the part did not break and fracture correctly did not occur in the simulation, the displacement of the impactor at the onset of part break is reasonably predicted as visible from the values reported in Table 4. 

In most cases, the force drop after the onset of failure is steeper in reality than in the simulation, as also observed in the test with the smaller impactor on the most expanded part (Figure 6). This might request further tuning of material card parameters, especially on the parameter named “FADEXP” previously mentioned. Tuning of material card parameters will be discussed in the next section. Furthermore, regarding the mentioned test where fracture did not occur, the simulation was stopped in correspondence with the displacement for which, in the experiment, the impactor sleigh hit a safety buffer which was not considered in the Finite Element model.

**Figure 8:**
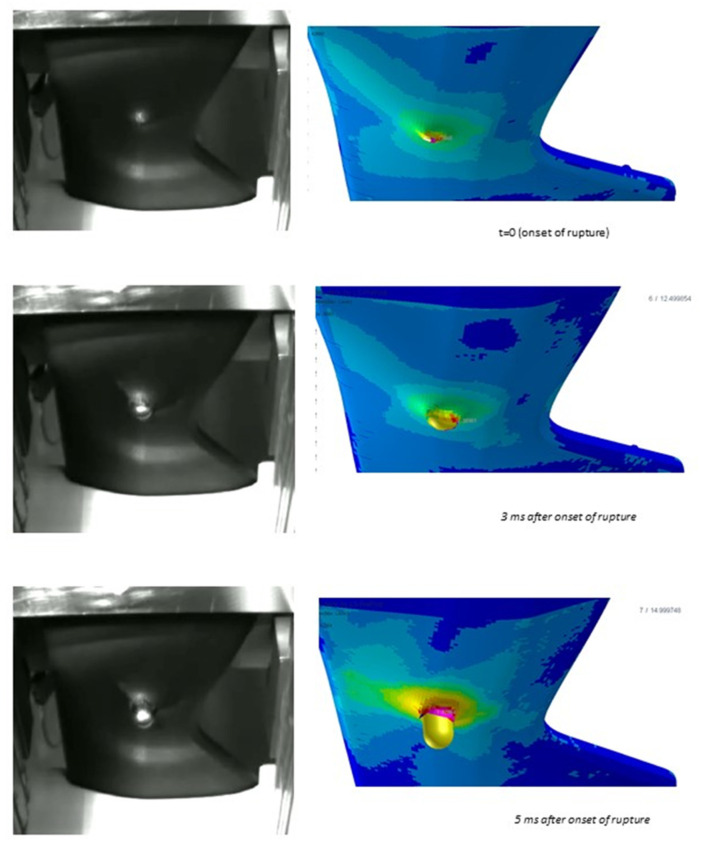
Comparison of fracture modes predicted by simulations (**right**) and observed experimentally (**left**) at different time instants during the impact sequence. Images refer to the 20 mm diameter impactor, impact speed of 3 m/s, and part thickness of 3.2 mm. Pictures from the experimental test provided by SMP GmbH., Bötzingen, Germany.

**Figure 9:**
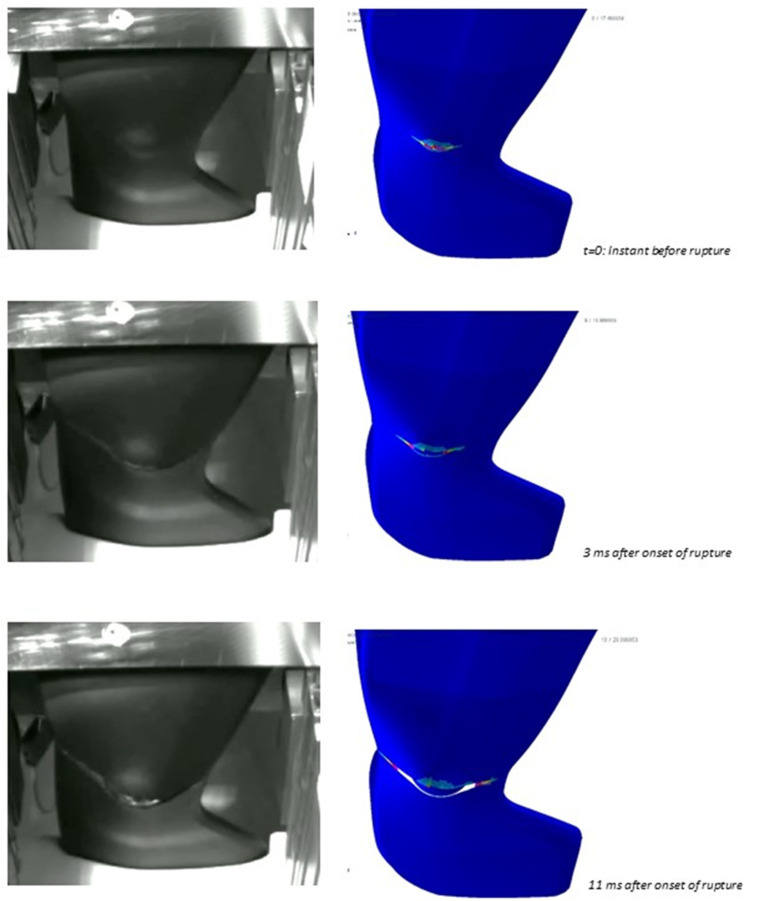
Comparison of fracture modes predicted by simulations (**right**) and observed experimentally (**left**) at different time instants during the impact sequence. Images refer to the 65 mm diameter impactor, impact speed of 2 m/s, and part thickness of 3.2 mm. Pictures from the experimental test provided by SMP GmbH, Bötzingen, Germany.

**Figure 10:**
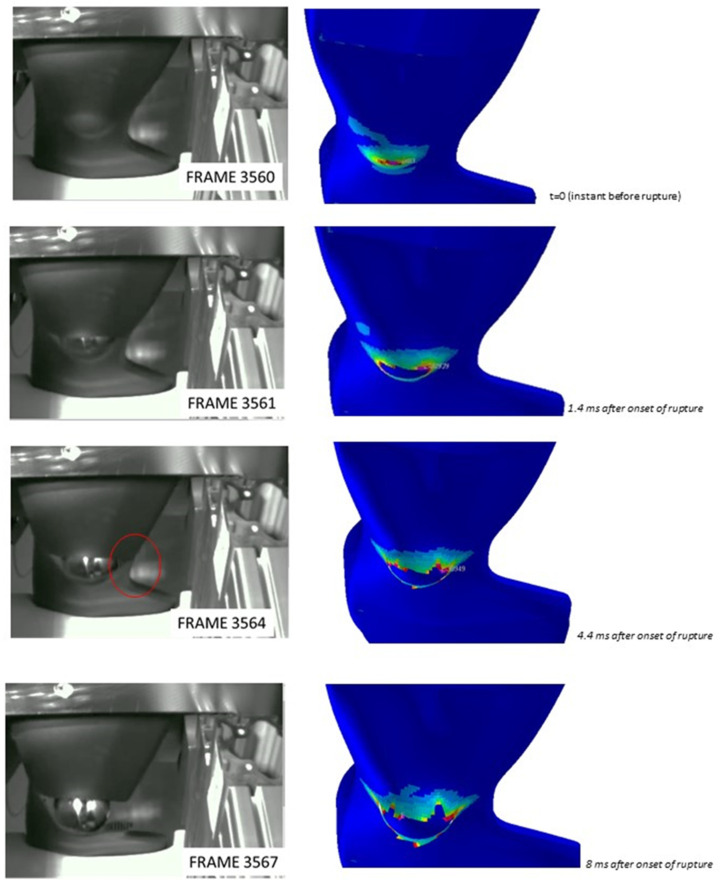
Comparison of fracture modes predicted by simulations (**right**) and observed experimentally (**left**) at different time instants during the impact sequence. Images refer to the 65 mm diameter impactor, impact speed of 4 m/s, and part thickness of 2.4 mm. Pictures from the experimental test provided by SMP GmbH, Bötzingen, Germany.

The validity of the failure criterion implementation based on damage is also assessed through the comparison of simulation results with images extracted from videos taken during the impact test, for which an extract is in Figure 8, Figure 9 and Figure 10; said images show where the fracture originates and how it propagates. In general, the numerical simulations were capable of discriminating situations where fracture occurs or not, in particular correctly predicting material failure when it happened in physical tests for the progressive increase of impactor speed from 2 m/s to 4 m/s, with parts produced with the same mold expansion.

As visible from Figure 8, Figure 9 and Figure 10, the onset of fracture in part under the impactor hit point is well captured by the simulations, and also the propagation is almost well predicted, with the exception of one load case, shown in Figure 10 (circled area; highest impact speed, large impactor, and highest expansion ratio). In some cases, fracture in simulations occurs for higher strains in comparison to the experimental tests. This could be related to lower elongation at break for the material at the strain rates involved in this application, which exceed those for which experimental tests were available. 

## 4. Discussion

It must be remarked again that the current model aims to provide a predictive tool that is especially useful for the early design stage. As such, its performance and limitations must be assessed considering this specific purpose. The main difficulty for foamed materials is that their mechanical characteristics are highly affected by the process conditions used for part manufacturing, which determine the foam structure and hence the mechanical behavior. The determination of material properties to be input in any simulation software is one critical step for the accuracy of the prediction. The method presented here aims to solve this problem through a dedicated material law calibration phase which needs experimental tests carried out on samples having a foamed structure similar to the one that will characterize the final part. Although this is not clearly known in detail “a priori”, it is well known that, in order to achieve the goals they are designed for, industrial parts need to have a regular and well-defined structure regarding bubble geometry, density, and distribution, which will not deviate significantly from what is generally expected. So, it is assumed that the foam structure on the final part will not differ much from the one that the designer devised, and the process conditions will try to achieve. Consequently, although in the validation case presented here, the material law parameters were derived from samples taken from the same parts used for the final validation, it is reasonable that for the application of this methodology, the calibration step can be carried out on samples extracted from other parts, provided that they are produced with the same process, using the same material, blowing agent and similar process parameters.

From this viewpoint, the significant advantage of the approach proposed here is that the material data to be given as input are measured on the compact grade, and no sophisticated and complex data need to be measured on foamed specimens, apart from the simple bending tests requested for the scaling coefficient calibration. As in [28], the rationale of this approach is that the role of the properties of the parent polymer material is crucial In determining the properties at the cellular stage, including failure. However, the cited paper [28] observes that very local strain rates occurring in the polymer of the cell walls can be at least one order of magnitude greater than those applied at the macro scale, which is, in fact, the strain rates occurring at the scale of the elements in a Finite Element analysis. This effect is believed to be a possible root cause of minor precision in the determination of fracture modes and localization of breaks on the part, as observed commenting Figure 10. However, it is remarked that in all the simulations, the occurrence of fractures on the part is well predicted with good correspondence between the experimentally recorded impactor displacement at the onset of fracture and the one predicted by the simulation. 

Based on the results and the assumption that the material structure can be modeled with a simple tree-layer sandwich structure, the results seem quite acceptable. The method already proved its validity in previous studies under static loading [19]; in the present study, the method showed quite a good prediction of the force vs. displacement curves in all the tests conducted with the small impactor, with some lack of accuracy only at high strains in the tests with the large impactor. In fact, as it is visible from Figure 8, for the tests with the small head impactor, the portion of the part which was damaged was only limited to the area under the impact point, while a larger portion of the part was damaged in the tests with the large impactor, as visible from Figure 9 and Figure 10. So, we assume that the main reason for the minor lack of accuracy of the present method at high strains is related to the assessment of the damage evolution, while the material law for the undamaged material and the procedure used for its parameter calibration provide quite satisfactory results. In fact, one possible cause that could be considered is the typical mechanical response of foamed structures under compression, where the collapse of the cells determines a plateau region in the overall stress-strain characteristics of the material [13]. However, a simple modification in the input data was tentatively explored, with the use of a different material law available in the solver, known as MAT_124 [16], which differentiates the behavior in tension and compression. The curves for describing the compressional behavior were derived from those from tensile testing by simply flattening the stress after the yield point. However, no significant difference in the simulation results was found. For brevity, these results are not displayed as the curves almost overlap with those displayed. 

It is believed that the damage in the impacted part may evolve in a different way from that occurring in the tests adopted to determine the damage curve for the model, which is measured under tensile loading. The damage mechanism in sandwich plates with polymer foam cores has been investigated in the literature [29,30], showing the importance of shear stresses developing in the core under bending loads. Similarly, it is recognized that stress triaxiality is relevant in the void growth in damaged materials during the “void coalescence phase”, which anticipates material failure [31]. Accordingly, damage in the core due to shear under bending could cause a reduced core support of the skins [30], hence reducing the bending moment. These aspects are not fully considered in the present model. Triaxiality effects on damage evolution are only addressed in a very simple way through Equation (3), but the 2D approach does not allow computing the shear stress within the part thickness. This is believed as a possible interpretation of the force overestimation observed for high strains under several testing conditions, especially for the larger impactor, due to the wider extension of the damaged area.

A further cause that could justify the observed discrepancy could be the heating due to viscoelastic effects, which could cause an increment of temperature in the material and subsequent material softening. However, thermal effects are already considered in the testing used to generate the material data and especially in the calibration phase for the determination of the scaling coefficient; however, this procedure was conducted on expanded specimens with a similar foam structure as the part, and nominal strain rates comparable to those occurring in the drop tests. Accordingly, it is believed that thermal effects are already present in the computational model.

In order to improve the accuracy of the prediction, considering all the above, a Finite Element model with solid elements should be employed, as a shell model is not suitable to model shear stresses in the core. Again, this would result in a more complex model and more relevant computational times, which conflicts with the need for an easy and quick tool for the designer in an early stage.

It is believed that further tuning of the material card and failure card parameters may lead to optimized results. However, this is not the purpose of this activity, which aims to supply a predictive tool. A dedicated set of experimental tests supplying information about rupture under different loading conditions, especially under shear and biaxial loading, could provide a further contribution to increasing the prediction accuracy. Unfortunately, state-of-the-art testing capabilities limit the reliability of such data as far as polymers are concerned, and this could be a critical development.

## 5. Conclusions

This paper proposes a simple numerical method to characterize and model the strain-rate-dependent behavior of foamed PP parts produced with core-back technology. The method, based on a three-layer model of the material, was validated on industrial parts subjected to an impact loading in a component test. A dedicated set of experiments was designed and then carried out for the specific purpose of method validation; it proved to provide a complete set of data, covering several aspects useful to highlight specific features of the model, including process-dependent material behavior, strain-rate dependent forces, part deformation and damage, and possible occurrence of fracture. The numerical model, which was easily implemented using the commercial finite element solver LS-DYNA, was able to reasonably predict the force-displacement characteristics on the impactor, including the damage and the occurrence of fracture of the material. It is believed that, although more accurate predictions could be obtained by a minor increase in the complexity of the model, the current accuracy of the prediction and the easiness of model implementation make it an interesting tool for part and component designers.

## Figures and Tables

**Table 1:** Basic properties of the compact grade used for this work measured at different strain rates.

Test Speed (mm/s)	Nominal Strain Rate (1/s)	Elastic Modulus (MPa)	Peak Stress (MPa)	Nominal Strain at Break (%)
0.1	0.01	1660	20.4	>130
10	1	1790	24.9	46
100	10	2030	28.3	38

**Table 2:** Results from bending tests at different speeds on specimens with different expansion ratios. Values are averaged on three tested specimens.

Nominal Thickness (mm)	Expansion Ratio	Peak Force (N)			Peak Stress (MPa)		
		Static	1 m/s	4 m/s	Static	1 m/s	4 m/s
2	1	11.6	17.7	18.6	26.0	39.5	42.1
2.4	1.4	16.0	24.2	26.3	23.7	36.2	39.4
2.8	1.7	19.9	28.6	31.5	21.6	32.3	35.0
3.0	1.9	20.1	30.9	34.9	20.3	31.2	34.7
3.2	2	21.7	32.0	40.0	18.9	29.2	34.6

**Table 3:** Model parameters are the input for the GISSMO card.

Expansion Ratio *r* (Range)	Elastic Modulus (MPa)	Damage at Failure	*n*	FADEXP
1	2030	0.49	0.95	0.015
1.0 < *r* < 1.2	1510	0.33	0.95	0.015
1.2 < *r* < 1.4	1250	0.14	0.95	0.015
1.4 < *r* < 1.6	1040	0.09	0.95	0.015
*r* > 1.6	710	0.08	0.95	0.015

**Table 4:** Impactor displacement (mm) at the moment of part fracture from experimental tests (Column “Test”) and from Finite Element Model (Column “FEM”).

Part Thickness (mm)	Impact Speed			
	2 m/s		4 m/s	
	Test	FEM	Test	FEM
2.4	None	None	54 mm	53 mm
3.0	26 mm	30 mm	28 mm	37 mm
3.2	28 mm	28 mm	29 mm	32 mm

## Data Availability

Not applicable.
